# Advanced electrical diagnostics for monitoring soil contamination: a laboratory-based assessment approach

**DOI:** 10.1038/s41598-026-37447-5

**Published:** 2026-02-17

**Authors:** Mostafa Moawad, Mohamed Gomaa, Ahmed Elshenawy, Alhussein Basheer, Adel Kotb

**Affiliations:** 1 Geology Department, Faculty of Science, Capital University (formerly Helwan University), Cairo, Egypt; 2 Geophysical Sciences Department, Head of Geophysical Exploration Group, National Research Centre, Cairo, Egypt; 3 Geophysical Exploration Department, Desert Research Centre, Cairo, Egypt

**Keywords:** Engineering, Environmental sciences

## Abstract

Soil contamination is one of the vital environmental challenges the world encounters. This study demonstrates the potential of electrical measurements in controlling and limiting the spread of contamination through soils. The current laboratory samples represent shale contaminated by oil containing polychlorinated biphenyls (PCBs). PCBs are serious risk to living organisms, particularly when they reach soils and aquifers. This study aims to show how electrical measurements can serve as a monitor for soil contamination and oil seepage. This contaminant is regarded as a semi-insulating material, whereas shale is supposed as a semi-conducting material. Different cases of shale samples (natural core, cracked core, and synthetic) and saturations (dry to fully saturated samples) with this contaminant were used. Electrical parameters (dielectric constant, conductivity, and impedance plane), for shale samples collected from Bani-Swef area, were measured. Therefore, samples (shale) were contaminated with oil at various contamination levels and concentrations to measure the electrical responses. These electrical measurements were performed at a frequency range starting from 1 mHz to 100 kHz. The results reveal that when levels of contamination increase, electrical conductivity and dielectric constant decrease for all sample cases. The contaminant reflects its insulator electrical characteristics. The laboratory measurements show reasonable results for natural and synthetic samples, while those for cracked samples exhibit abnormal behaviour, because the contaminant penetrates through the fractures and causes some distortion in the electrical readings. The electrical measurements show that it can be used as a preliminary screening step for monitoring soil contamination, particularly for oil seepage cases. It may offer a high-quality functional technique that is both cost-effective and non-invasive. This method may allow for a fast evaluation and assist as a massive environmental monitoring tool. This approach aligns with the sustainable development goals of Egypt vision 2023.

## Introduction

Soil contamination monitoring is an effective way to protect human health, the environment, and agricultural productivity. Contaminated soils may host hazardous substances including heavy metals, pesticides, and Persistent Organic Pollutants like polychlorinated biphenyls (PCBs), its chemical structure consists of two linked benzene rings in which hydrogen atoms can be substituted with chlorine atoms. The contaminated oil used in this study represents transformer oil potentially containing PCBs, mainly Aroclor 1254 congeners, which are commonly found in old electrical transformers at electricity companies^1,2^. PCBs are colourless and often have no smell. Because of their interactions with minerals, organic matter, and moisture content, oil containing PCB products can highly affect the electrical properties of soil. These contaminants can bio-magnify through the food chain, contaminate surface water, and seepage into groundwater aquifers. Chronic exposure to these contaminants can have health consequences that range from cancer to developmental problems and neurotoxicity. Early detection and continuous monitoring of pollutants in the soil allow for timely intervention, thereby preventing the spreading of contaminants and minimizing the cost and technical difficulty of any remediation effort. Thus, in addition to ensuring the safe use of land and the preservation of quality agricultural land for food security, effective monitoring also ensures that environmental laws are followed. Soil monitoring is also a key function for depicting the effectiveness of remediation techniques as policy and decision-making processes for natural resource protection for future generations. By knowing the level and consequences of soil contamination, communities will manage the risks and work for a cleaner and improved environment^3^.

The electrical characterization of porous materials is becoming more common in geologic investigations for environmental applications. Electrical approaches are non-destructive tools for monitoring soil contamination. Induced Polarization (IP) is one of the geophysical techniques increasingly utilized for monitoring soil contamination, particularly in detecting contaminants like Oil seepage. IP captures differences in dielectric constant and conductivity properties for different materials by measuring the sub-surface’s delayed electrical reaction by injecting electric current into the earth. These variations can be detectable through IP survey and provide critical insights into the presence, distribution, and contaminant concentrations^4–6^. Also, IP approach is a helpful non-invasive tool for risk-hazardous environmental monitoring since it may identify soil contamination^7–9^. By studying the changes in soil characteristics according to the contamination saturation, the electrical methods help identifying the contaminated hotspots and aquifers. PCBs tend to connect with soil rich in organic matter, changing its electrical characteristics, and IP can detect and display. This technology is very beneficial for contamination detection. Thus, using IP in soil contamination research helps to manage contaminated sites by oil seepage more precisely, effectively, and economically^6^.

The study of soil property changes is of great importance in environmental explorations. Due to its importance, many ancient and modern research studies have addressed it by studying the factors affecting changes in soil electrical properties in geophysical explorations. Studies have focused on the most important factors affecting these properties by the studying heterogeneity of samples^10–13^, the texture of samples^13^, the grain size of friable samples^14^, the effect of humidity and saturation^15^, the effectiveness of the salinity of samples, whether from the coastal aquifer^16^, and monitoring hydrocarbon leakage into the soil^8,17^, or by using different concentration levels of heavy metals in the soil affect the electrical properties^5,18^.

Numerous laboratory researches have indicated that IP impact is typically decreased when contaminants are present in water-saturated soil samples^19–^^28^ while other studies reach opposite results (increasing the IP effect) according to contaminant saturation^4,25,29–35^. Other trends have also been noted, in contrast to several laboratory investigations that have interpreted the relationship between non-aquas phase liquid contaminant concentrations and IP response as a linear relationship^6,23,25,33,36–40^. Despite the huge amount of laboratory research, it is not usually possible to compare the results of various studies due to sample preparation variations, different textures, and different contamination kinds^41^.

Different studies refer to using IP to support this method to detect the contaminated sites in addition to Spectral range because of their sensitivity to sample properties at pore scale levels such as porosity, permeability, grain size distribution, shapes of grains, sizes of grains, and pore throat size distribution^21,29,42–51^.

 The majority of previous studies have concentrated on sandy samples^23,25,33^, despite the increase of knowledge on IP technique applied to contaminated soils. On the other hand, shale samples have received little attention despite having unique mineralogical, low permeability, and high organic content that significantly affect electrical behavior. In the same context, there are no studies that have looked at how oil contamination levels specifically change the IP responses of different shale sample types (natural core, cracked core, and synthetic samples) in a controlled laboratory setting. This is a significant knowledge gap regarding the electrochemical mechanisms that control the interactions between contaminants and rocks in low permeability media. Thus, by performing systematic laboratory IP measurements on shale samples exposed to controlled oil contamination, the current study seeks to close this gap. The IP technique is useful for monitoring the effect of changing contamination concentration (oil) on the electrical characteristics^6^. In the same context, it is important to understand how these materials react with applied electric current. The IP technique uses measurements of electrical properties to reveal details about the properties of samples, particularly when it comes to contaminants like oil or metallic minerals^5^.

## Materials and laboratory methods

### Sample collection

**Fig. 1:**
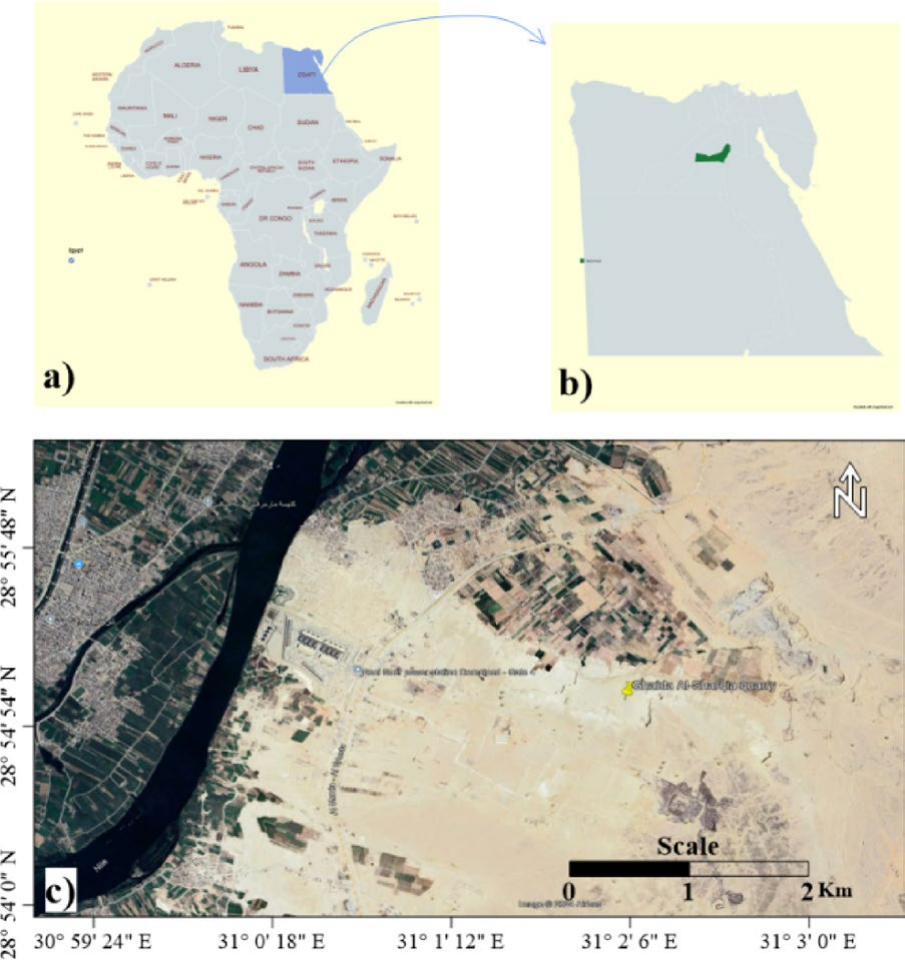
Location map of the study area, (**a**) spatial location of Egypt (hachured blue) relative to the Africa (gray) created by map chart, (**b**) spatial location of Beni-Swef district (green) relative to Egypt (gray) [a & b created by map chart, https://www.mapchart.net/], (**c**) spatial location of the collected studied samples area, which covered an area at west of Beni-Swef [the image source is https://www.google.com/earth/about/versions/ and full image were created and presented using Surfer^66^ V.15.5.382, https://www.goldensofware.com/products/surfer/.

Samples were collected from the Ghaida Al-Sharqia mine, Bani-Swef area, Egypt. The samples studied were collected from areas characterized by well-developed successions of shale deposits at latitude 28°55ʹ N and longitude 31°2ʹ E (Eastern part of the River Nile) (Fig. 1). These lithological features (successions) and sedimentary structures were measured, sampled, and reported by^52^. The mine of Ghaida Al-Sharqia (Bani-Swef area) is characterized by shale deposits in the Middle Eocene (El-Fashn Formation). Shale, limestone, and marl exposed at the Gabal Abiayd East El Fashn area usually make up this formation. Stratigraphically, Bani-Swef Formation is underlain by the El Fashn Formation, which unconformably covers the Qarara Formation (Middle Eocene)^52^. The site was selected because of the presence of a new electricity company which contains many transformers that uses oil and may contains the PCB products and may have some oil seepage. This oil seepage may affect the surrounding soil and human health. The general analyzed samples elements (trace and major) of studied samples were fully described by Soliman et al.^52^ and are listed in (Table 1).

**Table 1:** Major and trace element contents of the shales (after Soliman et al.^52^).

Element	Value (%)	Element	Value (ppm)
Sio_2_	46.09	Sr	254
Al_2_O_3_	15.08	Ba	208.8
CaO	5.3	V	122.4
MgO	1.73	Ni	79.5
Fe_2_O_3_	7.32	Cr	109.7
TiO_2_	0.95	Zn	118.3
P_2_O_5_	0.25	Cu	19.5
K_2_O	1.34	Zr	421.9
Na_2_O	1.97	Rb	51.9
SO_3_	0.14		

### Laboratory set-up and measurements

#### Experimental set-up

**Fig. 2:**
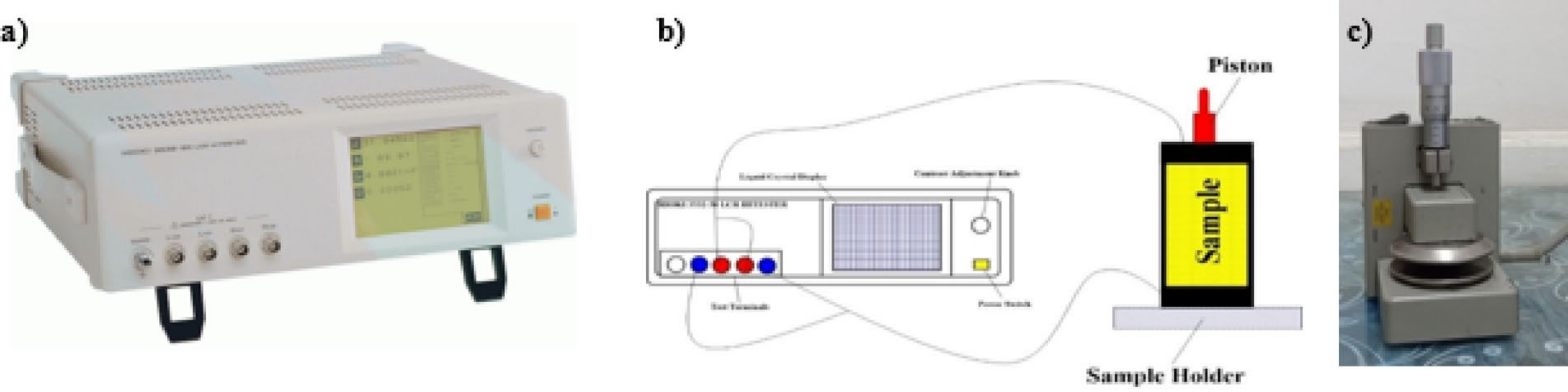
(**a**) The impedance analyzer, (**b**) the photographic diagram of the electrode system sample holder, and (**c**) the sample holder.

Electrical properties were measured using a cylindrical electrode design (Fig. 2a) to measure oil contamination of unsaturated shale (PCBs approximately 62 ppm). For synthetic Sample dimensions, according to sample holder dimension (thickness 25 mm, and diameter 40 mm), natural and cracked (thickness 15 mm, and diameter 6 mm).

The instrument used for this experiment is a Hioki 3522-50 LCR Hitester Impedance Analyzer (Fig. 2b) with a range of frequencies (1 mHz up to 100 kHz) across a voltage of around 1 V applied, and the instrument has been calibrated. The average value was obtained by measuring each point 64 times and four-electrode configuration was used to minimize electrode polarization and contact impedance (Fig. 2c), especially at low frequencies^32,36,53–55^.

**Fig. 3:**
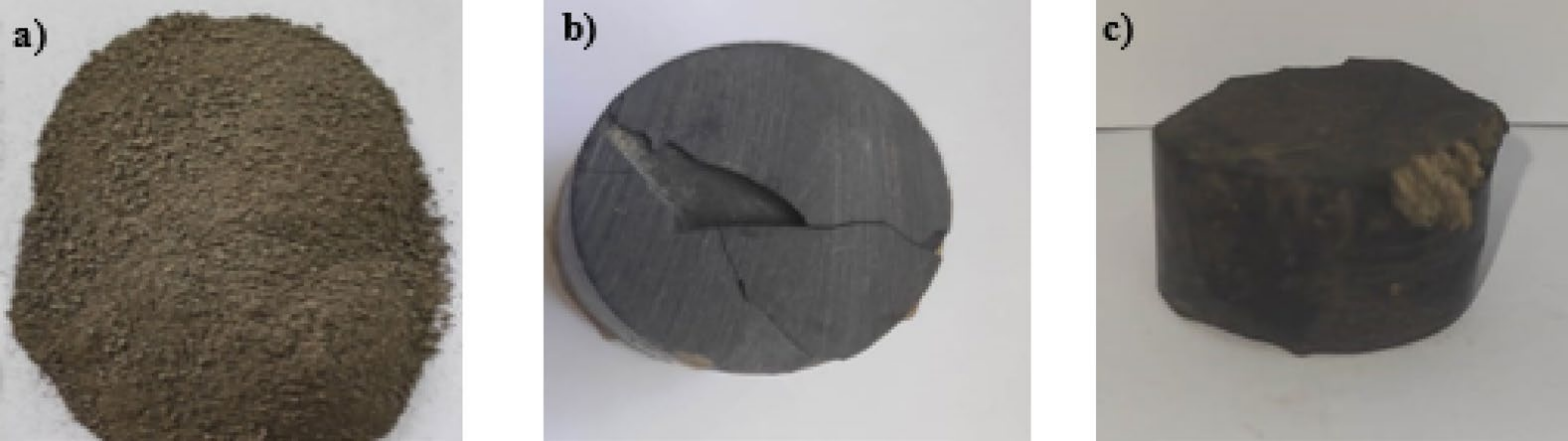
(**a**) Synthetic, (**b**) cracked, and (**c**) natural samples.

The collected samples were prepared by categorized into three different sample categories. The first category is synthetic samples (Fig. 3a), the cracked core samples (natural core samples but contain cracks) (Fig. 3b), and natural core samples (Fig. 3c).

**Fig. 4:**
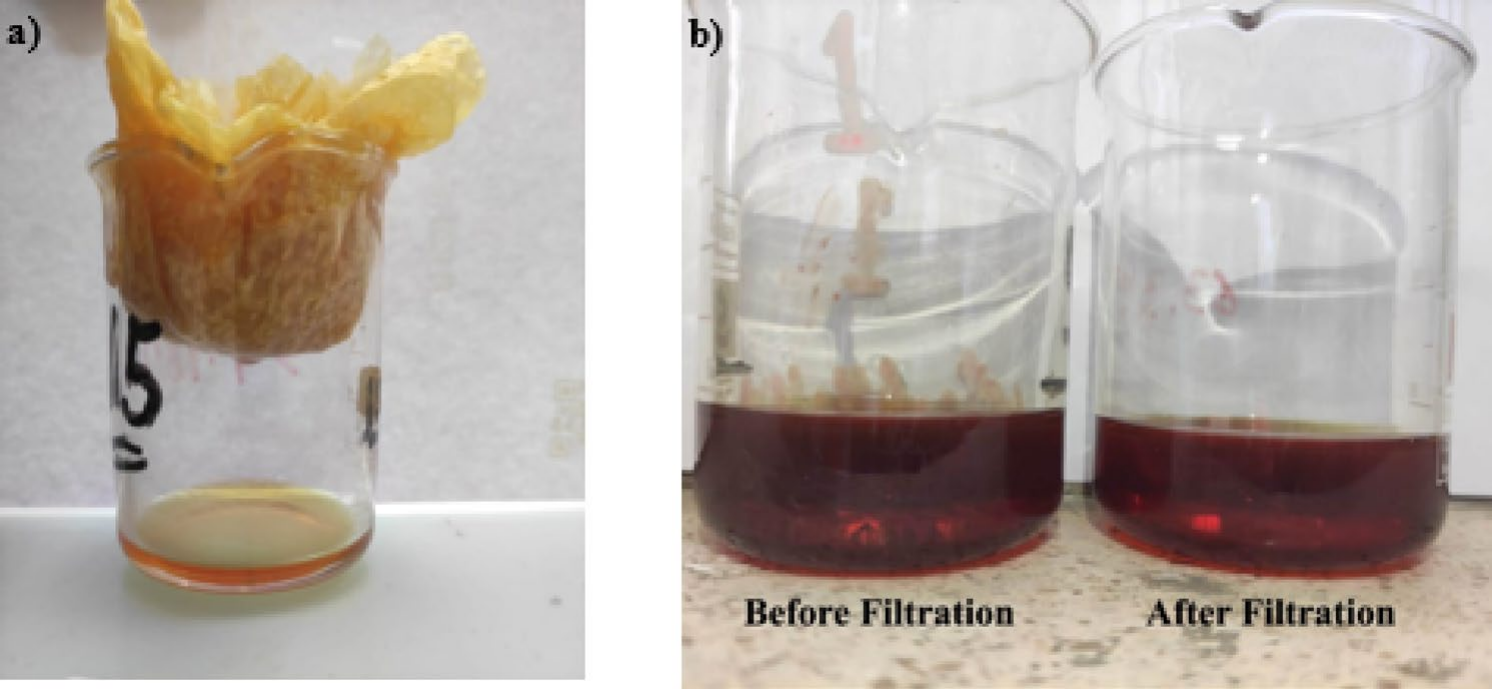
(**a**) Filtering process, and (**b**) differences between contaminants before and after filtering.

The contaminant oil containing PCBs around 62 ppm is used for this experimental work. In addition, to verify that the contaminant does not have any solid impurities (Fig. 4), which blocking the sample’s pores, the contaminant was filtrated.

**Fig. 5:**
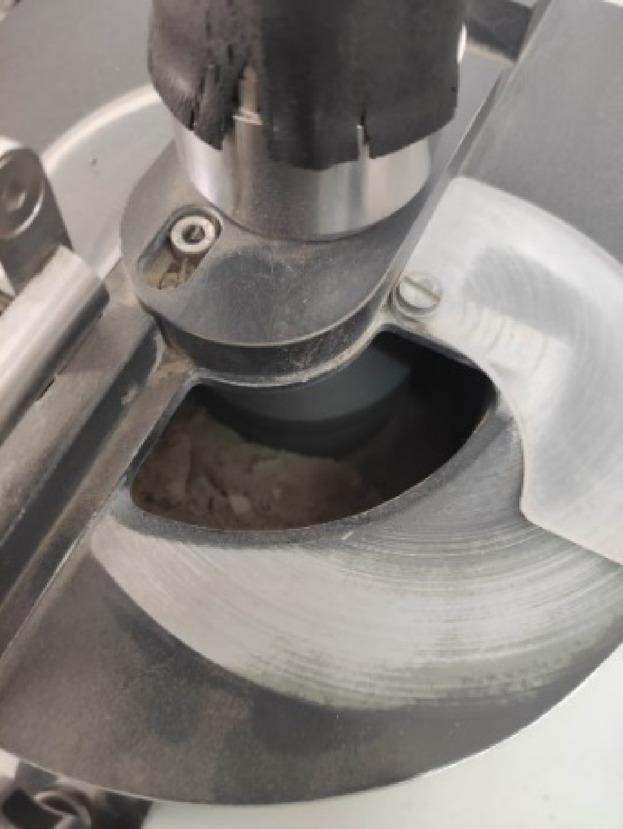
Agate mortar.

In the synthetic sample case, the rock sample became friable (Fig.3a) with agate mortar (Fig. 5). Then the sample was saturated with different amounts of contaminant (with pressure saturation was used) to get different saturation levels under the same pressure (4.5 Kb)^56^ to simulate field condition where the relative atmospheric humidity is ~ 64% and at room temperature (24 °C). The electrical measurements were measured after the determination of contaminant weight with increasing contaminant saturation. The percentage of contaminant concentration levels were calculated as

1 $$\:{W}_{s}=\left(\frac{{W}_{W}-{W}_{D}}{{W}_{D}}\right)\mathrm{*}100$$

where $\:{W}_{W}$ is the weight of the contaminated sample, and $\:{W}_{D}$ is the weight of a dry (uncontaminated) sample. The same procedures for the cracked (Fig. 3b) and natural samples (Fig. 3c) for the saturation by contaminant were applied. The porosity of studied samples (natural core ~15%, cracked core ~25%, and synthetic samples ~45%) were calculated as

2 $$\:\varnothing\:=\left(\frac{{V}_{p}}{{V}_{t}}\right)\mathrm{*}100$$

where, $\:{V}_{p}$ is the pore volume, and $\:{V}_{t}$ is the total volume of sample.

#### Computation of electrical parameters

The impedance analyzer instrument outputs are series and parallel capacitance and resistance (Cs, Cp, Rs, and Rp), respectively. Then, from these four parameters and the sample dimension as cross-sectional area (A) and thickness (d) of a sample, the complex impedance can be calculated from these equations:

3 $$\:{Z}^{\mathrm{*}}={Z}^{{\prime\:}}+i{Z}^{{\prime\:}{\prime\:}}$$

with

4 $$\:{Z}^{{\prime\:}}={R}_{s}\mathrm{*}\left(\frac{A}{d}\right)$$

5 $$\:{Z}^{{\prime\:}{\prime\:}}=\left(\frac{1}{\omega\:{C}_{s}}\right)\left(\frac{A}{d}\right)\:$$

where $\:{Z}^{{\prime\:}}$ and $\:{Z}^{{\prime\:}{\prime\:}}$ are real and imaginary (quadrature) components of impedance, respectively^41^.

The complex dielectric constant (permittivity)

6 $$\:{\epsilon\:}^{\mathrm{*}}={\epsilon\:}^{{\prime\:}}-i{\epsilon\:}^{{\prime\:}{\prime\:}}$$

must be calculated to measure the real and imaginary (quadrature) conductivity, and the complex component of the dielectric constant can be calculated as real and imaginary

7 $$\:{\epsilon\:}^{{\prime\:}}=\frac{{C}_{P}}{{C}_{0}}$$

8 $$\:{\epsilon\:}^{{\prime\:}{\prime\:}}=\frac{{G}_{P}.d}{\omega\:.A.{\epsilon\:}_{0}}$$

where

9 $${\text{Geometrical capacitance}}\;C_{0} = \left( {\frac{A}{d}} \right)\varepsilon _{0} \left( {\mathrm{F}} \right)$$

10 $$\varepsilon _{0} = {\mathrm{8}}.{\mathrm{85}} \times {\mathrm{1}}0 - {\mathrm{12}}\left( {{\mathrm{F}}/{\mathrm{m}}} \right),$$

11 $${\text{Parallel conductance}}\;G_{P} = \frac{1}{{R_{P} }}$$

12 $${\text{Angular frequency}}\;\omega = 2\pi f$$

The real part of complex conductivity can be measured by using this formula^43^

13 $$\:{\sigma\:}^{{\prime\:}}={\epsilon\:}^{{\prime\:}{\prime\:}}\mathrm{*}\omega\:\mathrm{*}{\epsilon\:}_{0}$$

#### Results and discussions

**Fig. 6:**
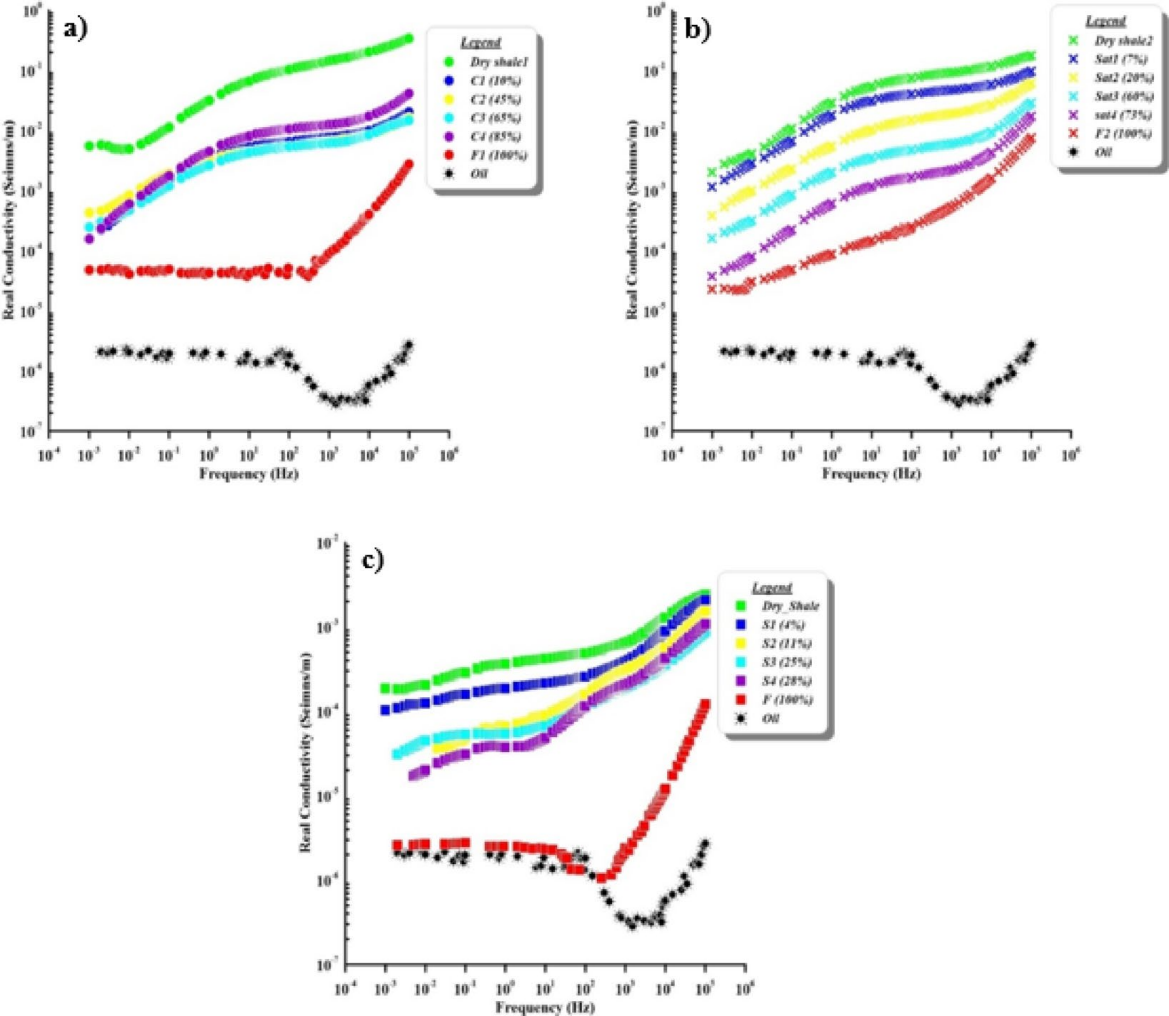
Variation in real conductivity with frequency according to increasing contaminant saturation for (**a**) cracked, (**b**) natural, and (**c**) synthetic samples.

The effects of contamination on the soil’s electrical characteristics were performed in this study. Frequency analysis of laboratory electrical measurements was presented through a range of 1 mHz up to 100 kHz. Hioki 3522-50 LCR Hitester Impedance analyzer was used to detect the electrical properties of different sample cases (cracked, natural, and synthetic). The results indicate that the contaminants affect the soil’s electrical properties, with increasing the contamination concentration, the electrical properties change rapidly. However, to study the effect of contaminants on soil, dry shale samples (Fig. 3) were taken and partially saturated with fractions of contaminant gradually from dry to different saturation levels (up to fully saturated). The experiment started by saturating natural core samples (Fig. 3c) by oil contaminant. With the increase of contamination levels, it was noticed that the conductivity of the sample did not decrease gradually (Fig. 6b). In this case, it was supposed that the contaminant may contain some solid impurities that block the pore spaces and do not penetrate the sample vertically. Accordingly, the contaminant was filtered (Fig. 4a) to ensure that it does not contain any solid impurities that block the upper pore spaces of the sample. Indeed, the purity of the contaminant was confirmed as it does not contain any solid impurities (Fig. 4b). Also, another sample with some fractures (cracked core) (Fig. 3b) was measured, and it was measured with the increase of contamination. It was observed that the contamination penetrates the sample deeply and moves through those cracks directly (does not penetrate the bulk sample). Moreover, trying to make a synthetic sample (Fig. 3a) by grinding some hand specimen samples. This step to avoid the heterogeneity of the samples and to be able to track the movement of the contaminant according to homogeneity. After some additions (oil saturation), it’s discovered that the contaminant developed some layers that blocked many contaminant particles from passing through the samples. There was very little movement of the oil between the pores.

It should be noted that the change in real conductivity (conduction paths in S/m) with increasing frequency as a function of contaminant saturation levels for the three cases are represented in Fig. 6 [cracked (Fig. 6a), natural (Fig. 6b), and synthetic (Fig. 6c) samples]. There are clear relationships between increasing contaminant saturation and conductivity. The conductivity values decrease from 7 × 10^− 3^ to 7 × 10^− 5^ S/m at cracked samples, and 2 × 10^− 3^ to 2 × 10^− 5^ S/m at natural samples, and 2 × 10^− 4^ to 2 × 10^− 6^ S/m at synthetic samples, while the value of pure contaminant conductivity is 1.8 × 10^− 6^ S/m. This conductivity value of the contaminant refers to an insulator material.

Börner et al.^29^ examine the different contamination (including oil as contaminant) for sand and shale synthetic samples then state that the conductivity decreases with increasing the oil contaminant. Besides, the conductivity values (Fig. 6) reflect logical trends between the three different sample cases but different ranges of conductivity values (Table 2) because of texture^57^. These trends suggest that with increasing contamination levels, there are significant decreases in conductivity values^31^. In the context, it is noticed that the fully saturated samples in different sample cases have two trends, one of them appears in cracked (Fig. 6a) and synthetic samples (Fig. 6c), following the same response of pure contaminant electrical characteristics, while the others appear with natural sample response (Fig. 6b) follows the same response that occurs in partially saturated samples. This can be interpreted as the synthetic fully saturated sample, semi-identical response due to the homogeneity of material with the contaminant and the response of cracked sample due to the accumulation of contaminant through the fractured exist in the sample, while the natural sample response according to the natural state of the sample. The mechanism of oil transport through the cracks due to gravity effect and the oil accumulate through these cracks then increase fluid pathways and accelerate contaminant migration, leading to local dielectric heterogeneity and unstable charge accumulation^58,59^. The polarization effects of oil particles make the soil less conductive at lower frequencies^17^. Because they are dielectric and comparatively non-conductive, these contaminants form an interface with the soil particles, which polarize when exposed to an electric field, especially at low frequencies. Polarization processes (considered noise signals), such as Maxwell-Wagner polarization, predominate at higher frequencies. As the frequency increases, these interfacial polarization effects decrease, causing the overall conductivity to decrease, especially in soils with high oil content. As well as, with changing grain sizes of different samples, there is no change in conductivity responses^22^. So, the finding results deal with different literatures as oil saturation increases the real conductivity decreases (e.g.^31,33,60^).

**Table 2:** The value range of different electrical parameters according to each different case.

Parameter	Sample case	Value range			Fig.
		From	To		
Real conductivity	Cracked core	7 × 10^− 3^ S/m	7 × 10^− 5^ S/m		6, a
	Natural core	2 × 10^− 3^ S/m	2 × 10^− 5^ S/m		6, b
	Synthetic	2 × 10^− 4^ S/m	2 × 10^− 6^ S/m		6, c
	Contaminant	1.8 × 10^− 6^ S/m			6
Dielectric constant	Cracked core	10^10^	8 × 10^8^		7, a
	Natural core	8 × 10^9^	2 × 10^8^		7, b
	Synthetic	8 × 10^8^	7 × 10^6^		7, c
	Contaminant	7 × 10^6^			7
Real impedance	Cracked core	2 × 10^2^ Ohm.m	2 × 10^4^ Ohm.m		8, a
	Natural core	8 × 10^2^ Ohm.m	5 × 10^4^ Ohm.m		8, b
	Synthetic	4 × 10^2^ Ohm.m	3 × 10^4^ Ohm.m		8, c
	Contaminant	7 × 10^5^ Ohm.m			8
Imaginary impedance	Cracked core	10^4^ Ohm.m	1 Ohm.m		9, a
	Natural core	10^4^Ohm.m	10 Ohm.m		9, b
	Synthetic	10^4^ Ohm.m	100 Ohm.m		9, c
	Contaminant	10^5^ Ohm.m			9

**Fig. 7:**
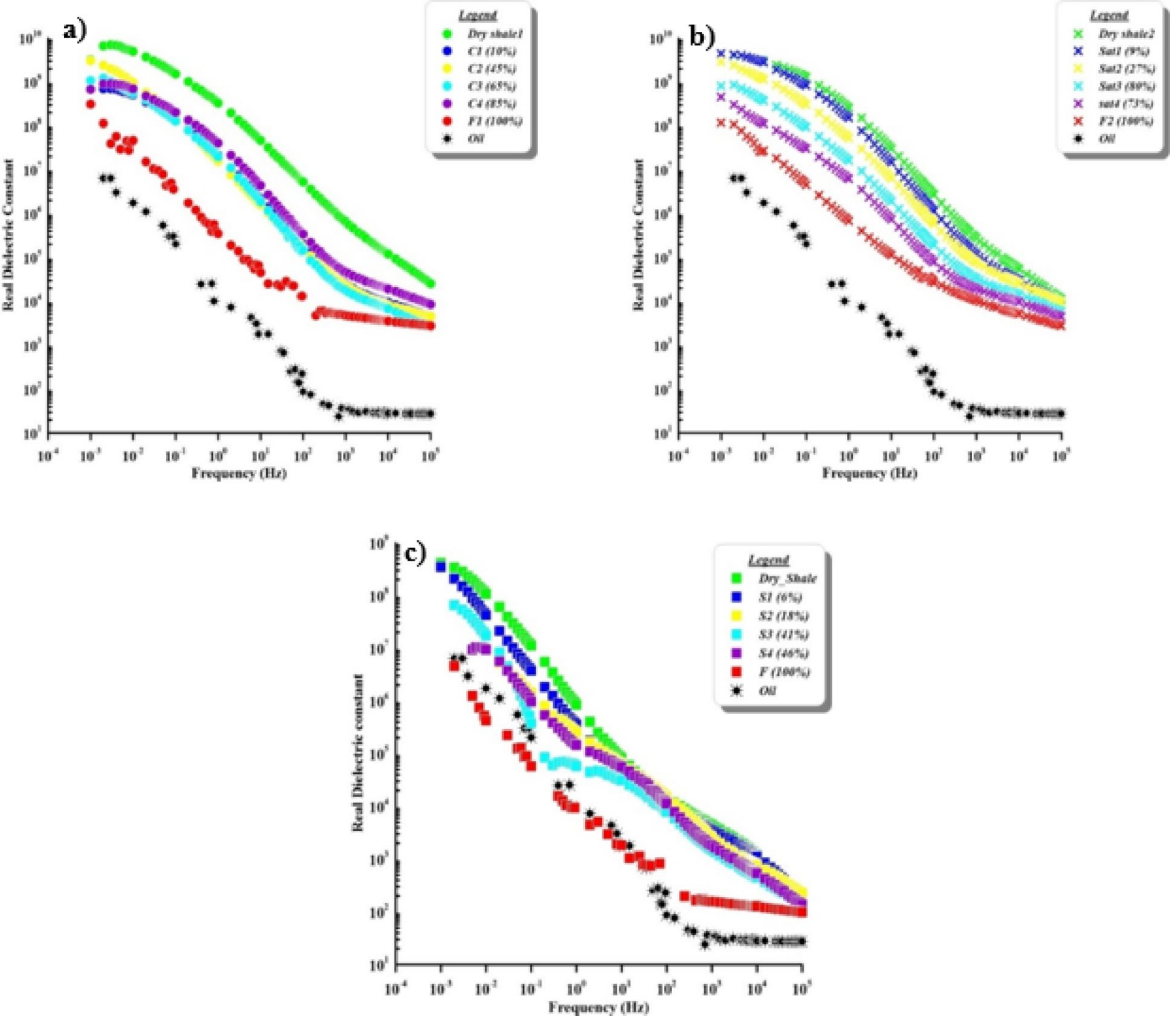
Variation in real dielectric constant (permittivity) with frequency according to increasing contaminant saturation for (**a**) cracked, (**b**) natural, and (**c**) synthetic samples.

The effect of increasing contamination levels on the dielectric constant characteristics for cracked, natural, and synthetic samples were showed in Fig. 7. There is a clear decrease in dielectric values by increasing the amount of contamination saturation within the whole range of frequencies. The dielectric constant values range from 1 × 10^10^ to 8 × 10^8^ in cracked samples, 8 × 10^9^ to 2 × 10^8^ in natural samples, and 8 × 10^8^ to 7 × 10^6^ in synthetic samples, while the value of pure contaminant is 7 × 10^6^.

The different level additions of contamination concentrations decrease the sample’s dielectric constant (Fig. 7) at low frequencies by nearly half a decade^8^, and the value decreases at relatively high frequencies. Relatively high dielectric constant values, at relatively high frequencies become frequency-independent after decreasing with increasing frequency. It notices a gentle decrease in natural sample cases (Fig. 7b), but decreases in the others are irregular. In samples, the dielectric constant tends to decrease as the frequency of the applied electric field increases. This is because charged particles (electric dipoles) cannot be rearranged with rapidly oscillating fields fast enough. This impact is particularly observed in oil contaminated soils because oil is non-polar and do not have the same polarizability as water molecules. The ability of the soil to store energy decreases with increasing oil concentration, which lowers the dielectric constant, particularly at higher frequencies. Particularly in soils with high oil saturation, the dielectric constant decreases as frequency increases, because polarization delays change in the electric field.

**Fig. 8:**
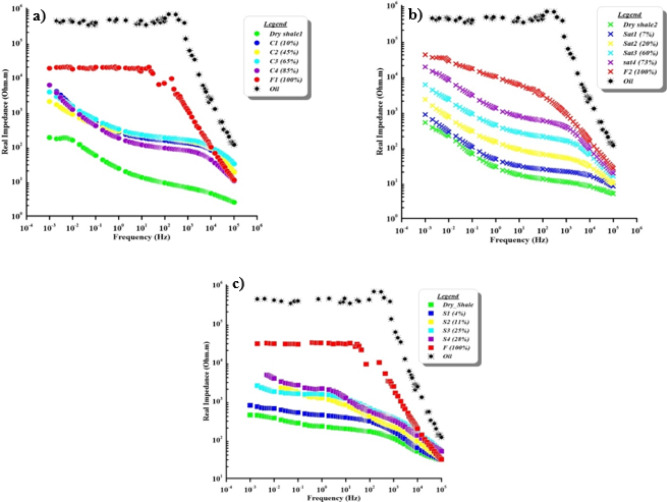
Variation in real impedance with frequency according to increasing contaminant saturation for (**a**) cracked, (**b**) natural, and (**c**) synthetic samples.

The effect of contaminant concentration on the real impedance (in Ohm.m) in the spectral range for the cracked, natural, and synthetic samples showed in Fig. 8. The real impedance decreases as frequency increases for all samples. The contaminant shows consistently high impedance values (7 × 10^5^ Ohm.m) across all frequencies compared to other samples. Samples with increasing contaminant saturation percentages (from dry to fully saturated) exhibit progressively lower impedance at corresponding frequencies. The real impedance values range from 2 × 10^2^ to 2 × 10^4^ Ohm.m for cracked samples, 8 × 10^2^ to 5 × 10^4^ Ohm.m for natural samples, and 4 × 10^2^ to 3 × 10^4^ Ohm.m for synthetic samples.

Besides, when the contaminant concentration increases, the real impedance increases. Long-range conduction pathways can form at low frequencies because charges have more time for movement. The electrode polarization mechanism dominates, contributing to lower impedance. At high frequencies, lower resistance results from charge carriers’ ability to follow the quickly fluctuating electric field and get conductive paths. Real impedance increases with frequency under contaminant saturation levels due to increased dielectric relaxation, decreased ionic mobility, and material microstructure changes. The electrical behaviour of soil is important to estimate material performance in different environments and determine the effects of contamination.

**Fig. 9:**
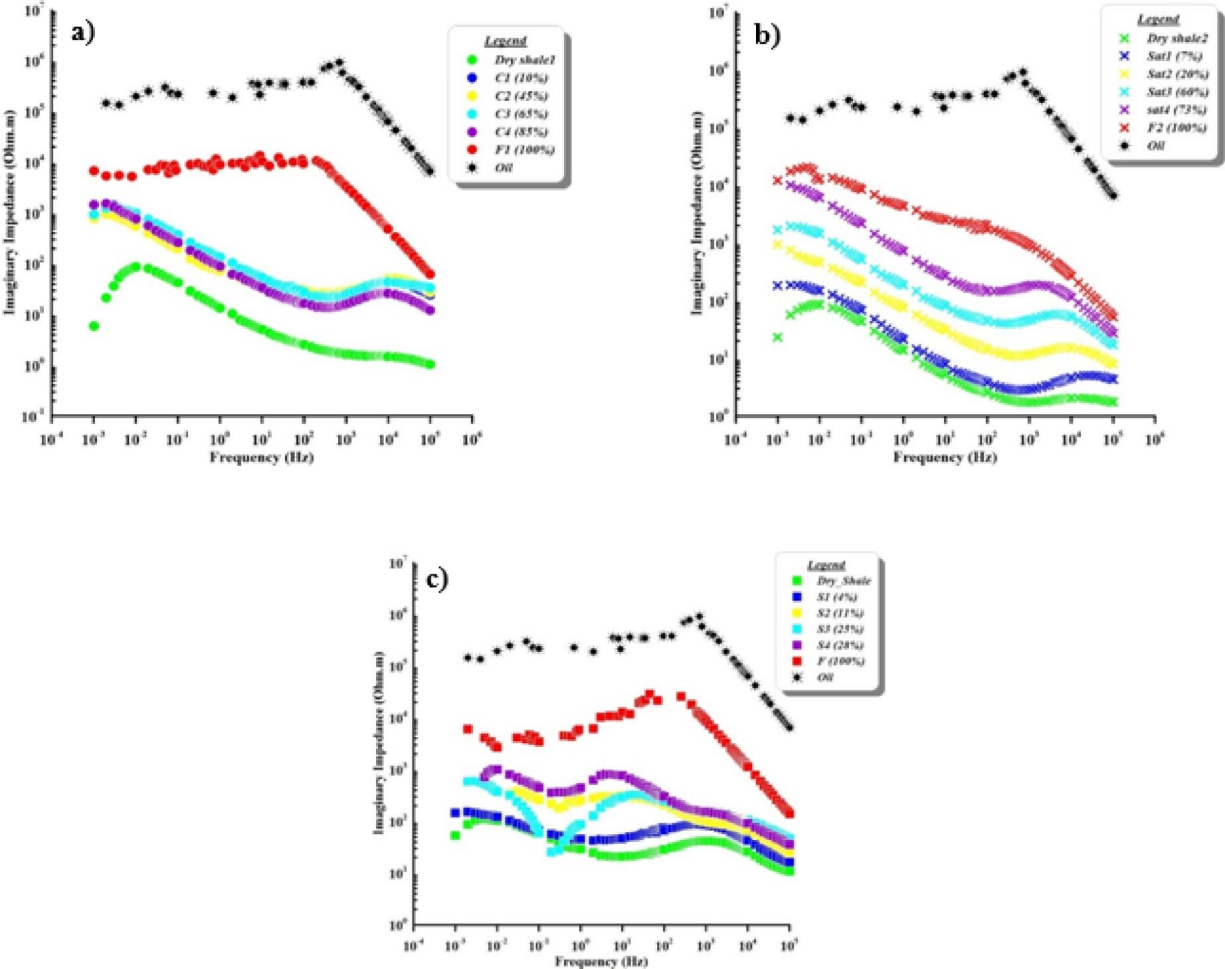
Variation in imaginary impedance with frequency according to increasing contaminant saturation for (**a**) cracked, (**b**) natural, and (**c**) synthetic samples.

The relationship between imaginary impedance (in Ohm.m) as a function of frequency for cracked, natural, and synthetic samples with different contaminant concentration levels are illustrated in Fig. 9. Dry shale exhibits higher impedance compared to partially saturated samples at lower frequencies. The pure contaminant has a significant imaginary impedance value (10^5^ Ohm.m). The range of the cracked sample case is from 10^4^ to 1 Ohm.m, the natural sample case is from 10^4^ to 10 Ohm.m, and the synthetic sample is from 10^4^ to 100 Ohm.m.

The different responses among different samples emphasize the influence of geological and structural properties on their electrical behavior. In the same context, the cracked samples (Fig. 9a), higher imaginary impedance values are observed at low frequencies, which can be attributed to interfacial (Maxwell–Wagner) polarization caused by the large contrast in electrical properties between the non-conductive oil and conductive shale^61–63^. Fractures increase effective porosity and facilitate conductive pathways, making the impedance response highly sensitive to changes in oil saturation^64^. Besides, at the natural samples (Fig. 9b), characterized by finer porosity and a tighter structure, the response appears more gradual, with an overall decrease in imaginary impedance as saturation increases. This suggests limited infiltration and distribution of oil within the fine pore spaces, which reduces its impact on the electrical properties. Laboratory dielectric relaxation studies of partially saturated shales verify this behavior^36^. Conversely, the synthetic samples (Fig. 9c) exhibit smoother and more uniform behavior, reflecting a homogeneous distribution of saturation within the sample. This indicates that synthetic materials can serve as idealized models for studying the electrical response of oil-contaminated shale, although they may not fully capture the complexities of natural cases. A recent study on synthetic shale–sand mixtures confirm the consistent impedance trends and dielectric dispersion observed in such engineered media^51^.

The Nyquist plot (Argand plane), which depicts the relationship between real impedance (on the X-axis, in Ohm.m) and imaginary impedance (on the Y-axis, in Ohm.m). This relationship is widely used in electrochemistry, materials science, and geophysics to analyze electrical and dielectric properties. This plot represents different electrical responses related to the homogeneity of the materials. One of these responses is a skewed arc which represents how the medium refers to insulator material, then changed to one semicircle which reflect a semi-insulating material. In addition to the diameter of this semicircle giving information about the electrical characteristics of the materials. Another shape is the two semicircles due to the bulk mixture and sample properties. The first semicircle (at high frequencies) reflects properties of the bulk impedance (impedance of the main material), and the second semicircle (at low frequencies) reflects the interface impedance (impedance of the surrounding material). On the other hand, that the two axes (the X-axis and the Y-axis) must be the same scale to show the semicircular curve. It is commonly used to analyze the electrical properties of materials, such as shale, in terms of their resistive and capacitive behaviour.

**Fig. 10:**
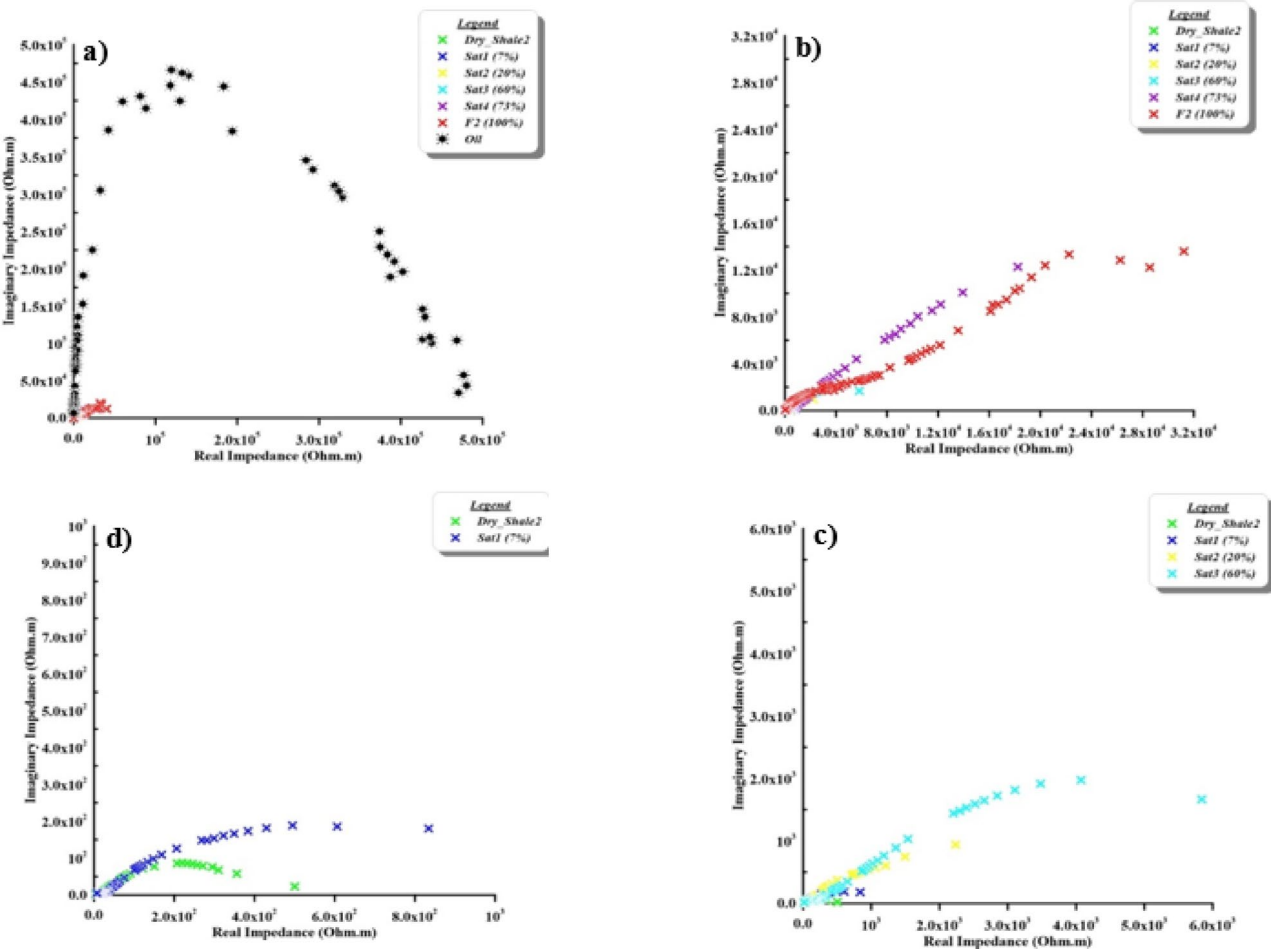
The argand (impedance/Nyquist) plane of natural samples as a function of contaminant concentration. The first panel presents a zoomed-in view while the subsequent panels are displayed at progressively larger scales to capture the full impedance response across the entire concentration range.

Nyquist plot of the natural samples is presented in Fig. 10. The contaminant appears to form a distinct wide semicircular curve (Fig. 10a) forms a distinct semicircular curve and represents a high resistance and capacitance nature. To be able to see the other samples, the two axes were rescaled (Fig. 10b–d). Grains at natural shale samples (Fig. 10), with increasing contaminant concentration, tend to make a cluster at the lower left of the plot, showing low impedance values. Dry shale is close to the origin, indicating very low impedance compared to contaminants. As contaminant concentrations increase, the impedance values move closer to the origin, implying that higher fluid content reduces both the resistive and capacitive impedance components^8^. There are bulk (properties of the material) and interface (properties of surrounding contamination) impedances that appear in partially saturated samples (Fig. 10b and c). The bulk (at high frequency) and interface (at low frequency) impedances are clear as two semicircular curves (Fig. 10b). From this point of view, one semicircular curve appears in the dry and low concentration levels with contaminant (Fig. 10c, and d).

**Fig. 11:**
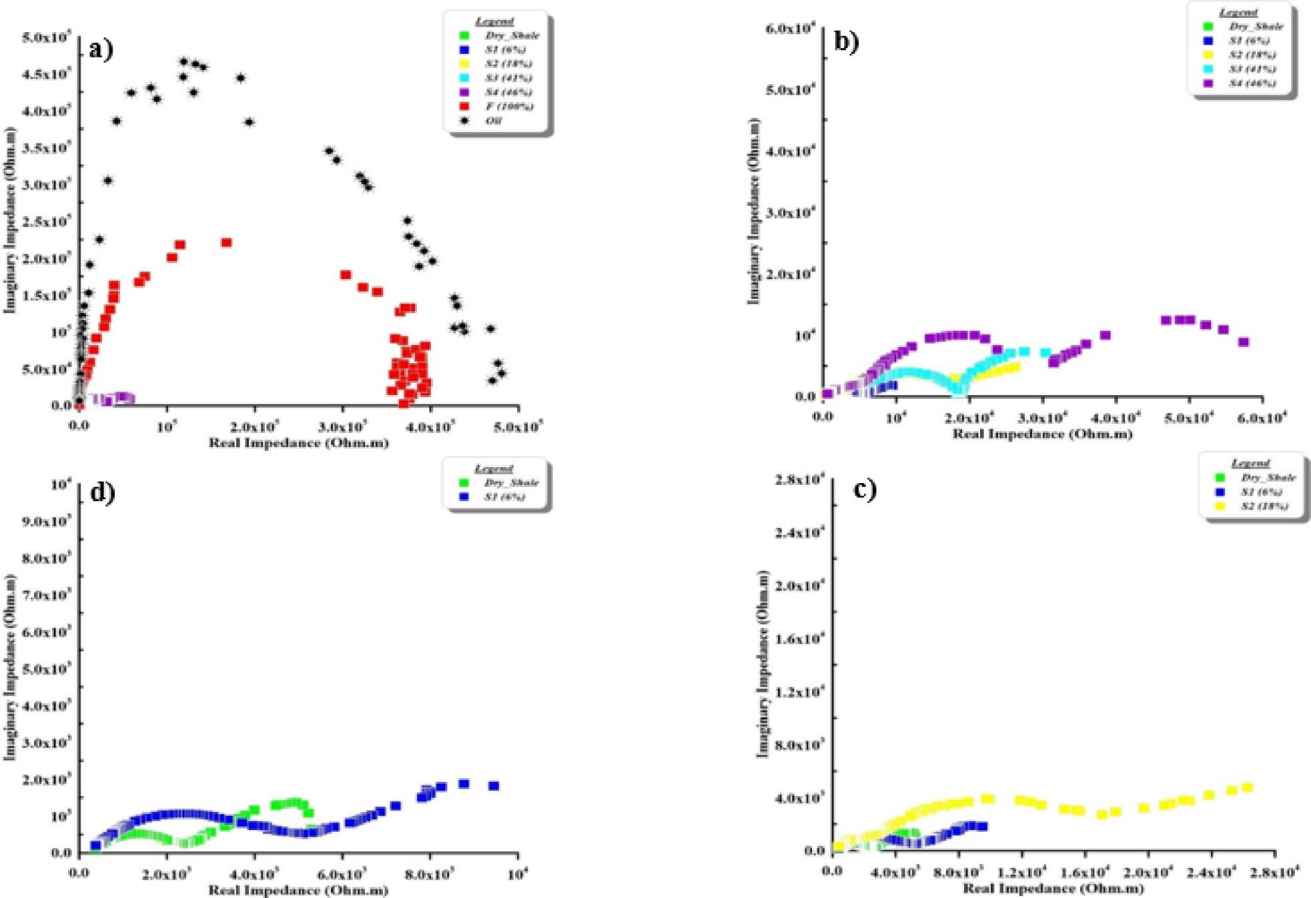
The argand (impedance/Nyquist) plane of synthetic samples as a function of contaminant concentration. The first panel presents a zoomed-in view while the subsequent panels are displayed at progressively larger scales to capture the full impedance response across the entire concentration range.

**Fig. 12:**
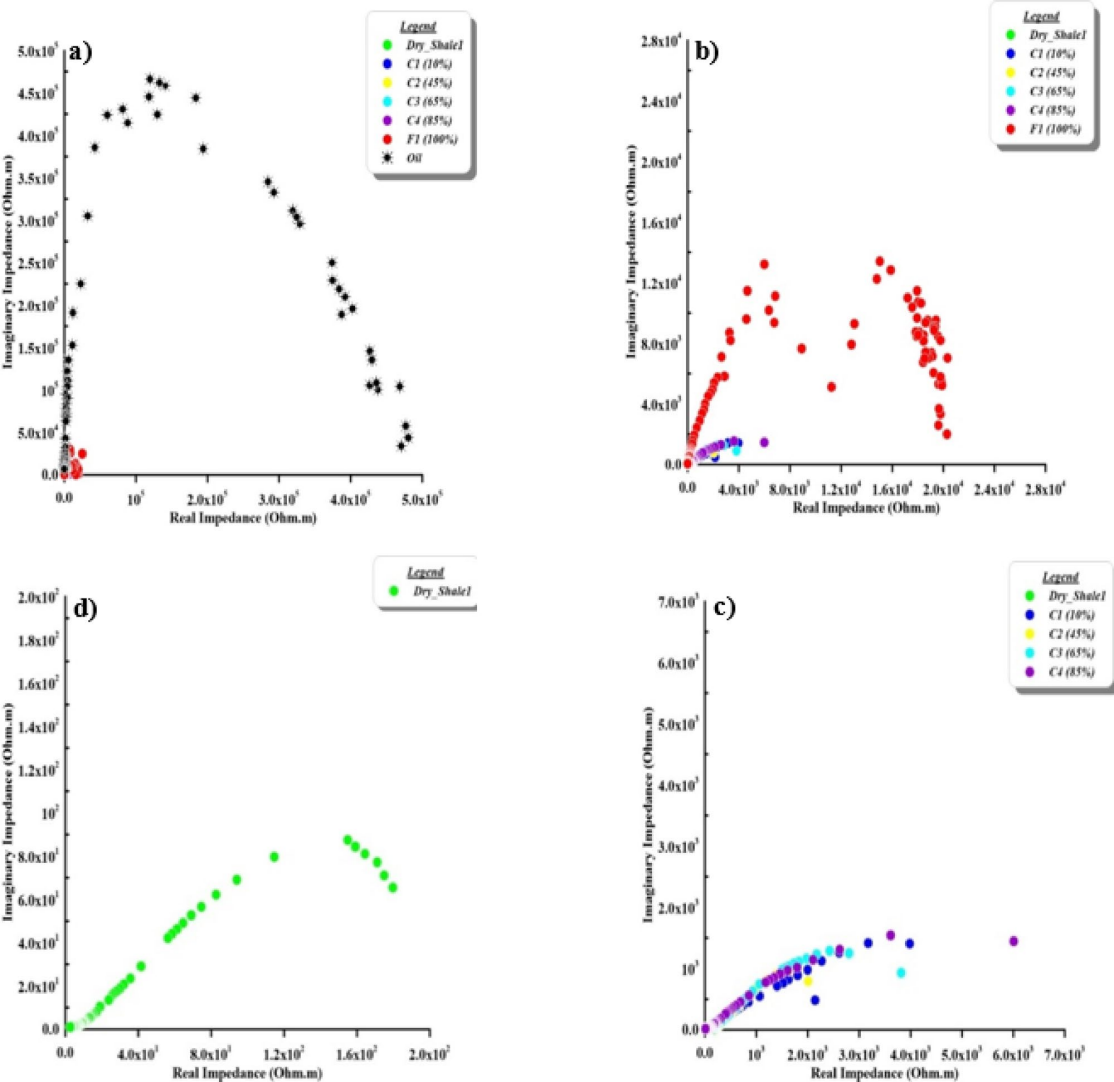
The argand (impedance/Nyquist) plane of cracked samples as a function of contaminant concentration. The first panel presents a zoomed-in view while the subsequent panels are displayed at progressively larger scales to capture the full impedance response across the entire concentration range.

Impedance plane of the synthetic samples case is presented in Fig. 11. It clearly shows the bulk and interface impedances in all samples as two semicircular curves. This is due to completely homogenous material with contaminants. On the other hand, in the cracked samples case doesn’t show the soil behaviour bulk and interface impedances due to the accumulate the contaminant through cracks (Fig. 12).

**Fig. 13:**
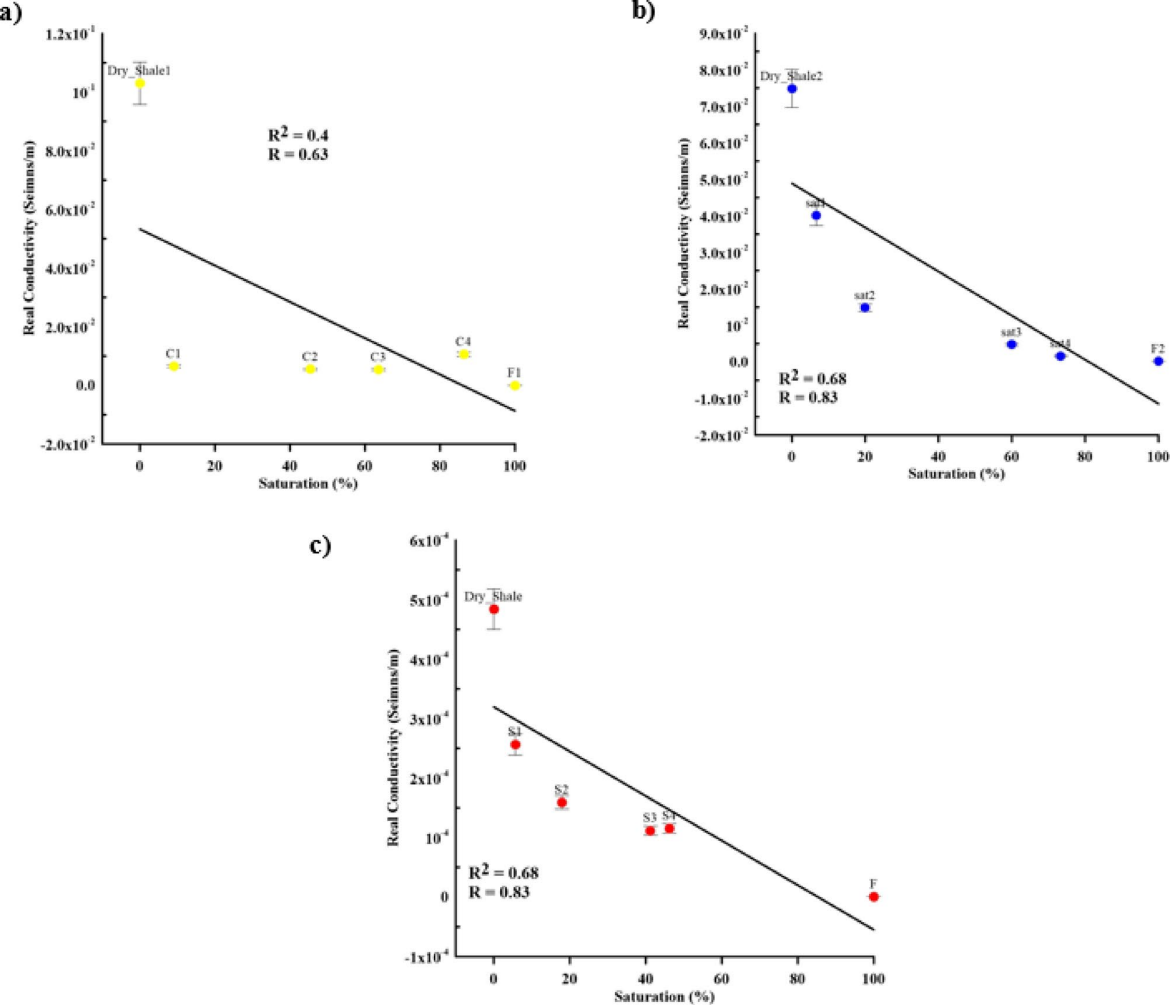
The effect of contaminant concentration on real conductivity according to (**a**) cracked (yellow circles), (**b**) natural (blue circles), and (**c**) synthetic (red circles) samples at 100 Hz with error bars.

As a reference for highlighting the changes of the IP signal, 100 Hz was selected in this experiment^65^. The trend of real conductivity values over different contaminant concentration levels with error bars represent standard deviation is showed in Fig. 13. There are good inverse linear relationships between conductivity values and contaminant concentration levels with natural (*R* = 0.83, R^2^ = 0.68) and synthetic (*R* = 0.83, R^2^ = 0.68) samples, while the inverse linear relationship with cracked samples (*R* = 0.63, R^2^ = 0.4) was poor.

**Fig. 14:**
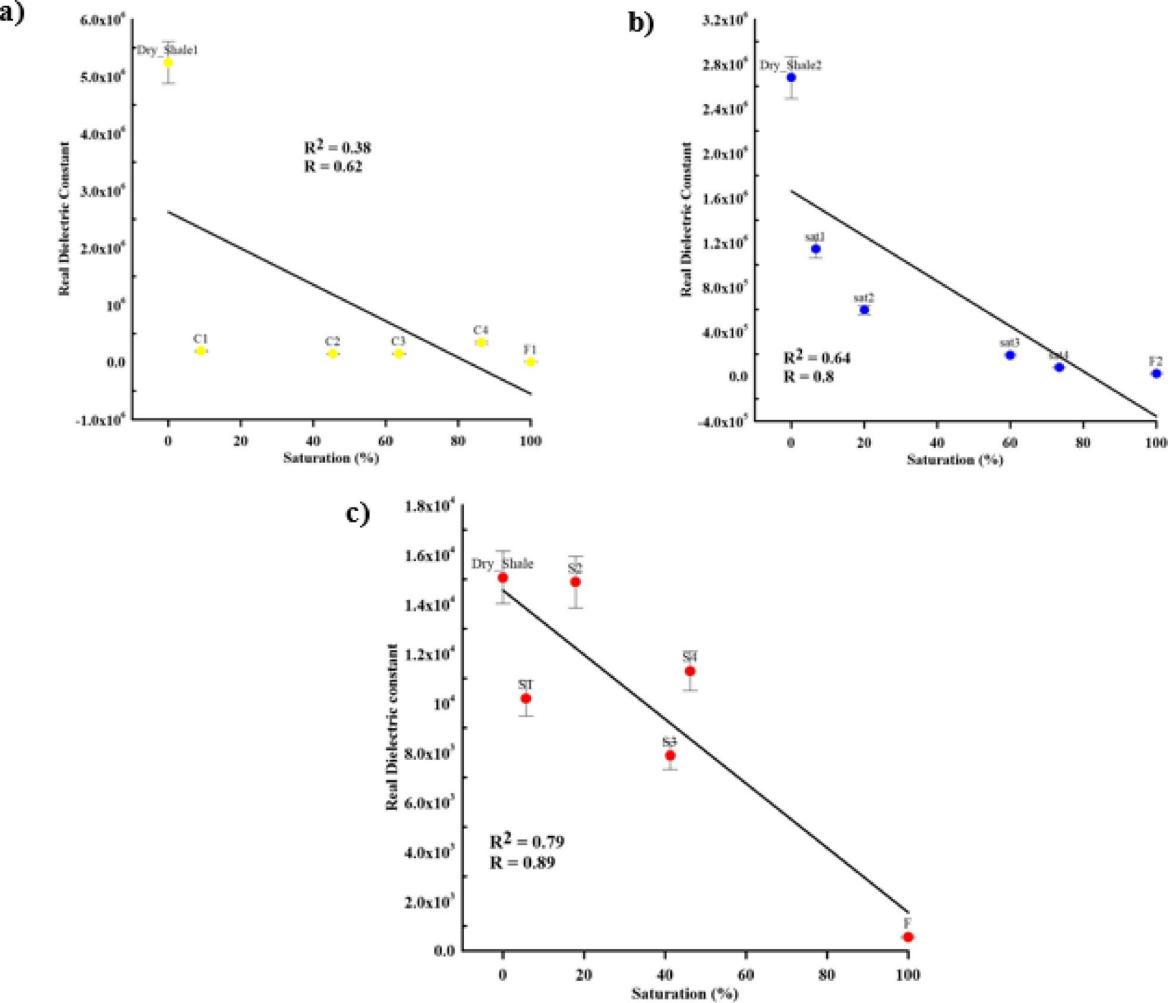
The effect of contaminant concentration on real dielectric constant according to (**a**) cracked (yellow circles), (**b**) natural (blue circles), and (**c**) synthetic (red circles) samples at 100 Hz with error bars.

The change in dielectric constant values over different contaminant concentration levels with error bars represent standard deviation is presented in Fig. 14. There are good inverse linear relationships between dielectric constant values and contaminant concentration levels with natural (*R* = 0.8, R^2^ = 0.64) and synthetic (*R* = 0.89, R^2^ = 0.79) samples, while the inverse linear relationship with cracked samples (*R* = 0.62, R^2^ = 0.38) was poor. General trends exist, such as conductivity and dielectric constant decreases with increasing contamination concentrations. This trend occurs as the contaminant particles block the conduction paths that current pass through. According to these relationship results, there are good relations in the case of natural and synthetic samples due to the natural and homogeneity of samples while there are poor relations in the case of cracked samples because of the contaminant accumulating through the sample’s fractured zones.

#### Conclusion

This work aims to monitor the effect of contamination (oil seepage) on soil using electrical measurement techniques. The experiment designed by using different samples to differentiate between all these cases on the laboratory scale. It was confirmed that there were no solid impurities in the contaminant that may affect the measurements. The study shows the following results:

- The electrical measurements of the fully saturated fractured sample follow the same response of the pure contamination and notice that it appears as a transition zone between the natural sample and synthetic sample.
- The dielectric constant shows storage energy, and with increasing the contaminant amount, there is a decrease in the energy stored.
- There is a good inverse linear relation between electrical properties and contamination concentration levels in the case of natural and synthetic samples, and a poor relation in the cracked samples case due to the transmissivity of oil through fractures.
- When the saturation of contaminants increases, the real conductivity and dielectric constant decrease.
- Based on electrical measurements, it can monitor the change in electrical characteristics according to the seepage of contamination.
- Measurements were able to differentiate between fractured and non-fractured samples.

The current study helps to better understand the behaviour of contaminants according to their electrical characteristics. It helps also when studying the geoelectrical experiment inside groundwater aquifers to monitor contaminants oil contained PCB products seepage. In future work, it recommends applying this study in the field and make a correlation between field and lab scales.

## Data Availability

The datasets used and/or analysed during the current study available from the corresponding author on reasonable request.

## References

[1] Breivik, K., Sweetman, A., Pacyna, J. & Jones, K. Global production and consumption. *Sci. Total Environ.* **290** (1–3), 181–198. 10.1016/s0048-9697(01)01075-0 (2002). Towards a global historical emission inventory for selected PCB congeners — a mass balance approach1. 10.1016/s0048-9697(01)01075-0 12083709

[2] Erickson, M. D. & Kaley, R. G. Applications of polychlorinated biphenyls. *Environ. Sci. Pollut. Res.* **18** (2), 135–151. 10.1007/s11356-010-0392-1 (2011). 10.1007/s11356-010-0392-1 20848233

[3] Grifoni, M. et al. Soil remediation: towards a resilient and adaptive approach to deal with the Ever-Changing environmental challenges. *Environments* **9** (2), 18. 10.3390/environments9020018 (2022).

[4] Cassiani, G., Kemna, A., Villa, A. & Zimmermann, E. Spectral induced polarization for the characterization of free-phase hydrocarbon contamination of sediments with low clay content. *Near Surf. Geophys.* **7** (5–6), 547–562. 10.3997/1873-0604.2009028 (2009).

[5] Gomaa, M. M., Melegy, A., Metwally, H. & Hassan, S. Geochemical and electrical characterization of heavy metals in contaminated soils. *Heliyon* **9** (6), e04954. 10.1016/j.heliyon.2020.e04954 (2020). 10.1016/j.heliyon.2020.e04954 PMC7502585 32995631

[6] Johansson, S., Fiandaca, G. & Dahlin, T. Influence of non-aqueous phase liquid configuration on induced polarization parameters: conceptual models applied to a time-domain field case study. *J. Appl. Geophys.* **123**, 295–309. 10.1016/j.jappgeo.2015.08.010 (2015).

[7] Deng, Y., Shi, X., Revil, A., Wu, J. & Ghorbani, A. Complex conductivity of oil-contaminated clayey soils. *J. Hydrol.* **561**, 930–942. 10.1016/j.jhydrol.2018.04.055 (2018).

[8] Gomaa, M. M., Elnasharty, M. M. M. & Rizzo, E. Electrical properties speculation of contamination by water and gasoline on sand and clay composite. *Arab. J. Geosci.* **12** (18). 10.1007/s12517-019-4767-4 (2019).

[9] Kang, X. et al. Characterization of DNAPL source zones in clay-sand media via joint inversion of DC resistivity, induced polarization and borehole data. *J. Contam. Hydrol.* **258**, 104240. 10.1016/j.jconhyd.2023.104240 (2023). 37683375 10.1016/j.jconhyd.2023.104240

[10] Abd El Aziz, E. A. & Gomaa, M. M. Electrical properties of sedimentary microfacies and depositional environment deduced from core analysis of the syn-rift sediments, Northwestern shore of Gulf of Suez. *Egypt. J. Petroleum Explor. Prod. Technol.* **12**, 2915–2936. 10.1007/s13202-022-01484-3 (2022).

[11] Boyle, M. H. The electrical properties of heterogeneous mixtures containing an oriented spheroidal dispersed phase. *Colloid Polym. Sci.* **263** (1), 51–57. 10.1007/bf01411248 (1985).

[12] Gomaa, M. M. & Abd El Aziz, E. A. Depositional environment, petrophysical evaluation and electrical properties of zeit Formation, Northwestern shore of Gulf of Suez, Egypt. *J. Earth Sci.* **35** (5), 1720–1737. 10.1007/s12583-023-1858-7 (2024).

[13] Gomaa, M. M. & Sedeek, H. Prediction of heterogeneity and anisotropy of oxidized pyrite crystals using electrical measurements. *NRIAG J. Astron. Geophys.* **10** (1), 244–257. 10.1080/20909977.2021.1913394 (2021).

[14] M Gomaa, M. Grain size effect on electrical properties of dry friable sand. *Eur. Phys. Journal: Special Top.*10.1140/epjs/s11734-022-00667-7 (2022).

[15] Moiz, S. A., Ahmed, M. M. & Karimov, K. S. Effects of temperature and humidity on electrical properties of organic semiconductor orange dye films deposited from solution. *Jpn. J. Appl. Phys.* **44** (3R), 1199. 10.1143/jjap.44.1199 (2005).

[16] Ammar, A. I., Gomaa, M. M. & Kamal, K. A. Applying of SP, DC-Resistivity, DC-TDIP and TDEM soundings in high saline coastal aquifer, Heliyon, **7**, Issue 7, pp. 1–20. (2021). 10.1016/j.heliyon.2021.e07617 10.1016/j.heliyon.2021.e07617 PMC8340133 34381891

[17] Capozzoli, L., Giampaolo, V., De Martino, G., Gomaa, M. M. & Rizzo, E. Geoelectrical measurements to monitor a hydrocarbon leakage in the aquifer: simulation experiment in the lab. *Geosciences* **12** (10), 360. 10.3390/geosciences12100360 (2022).

[18] Xie, X. et al. Complex resistivity spectrum of pollutant soils with low-concentration heavy metals. *Heliyon* **9** (10), e20541. 10.1016/j.heliyon.2023.e20541 (2023). 37800064 10.1016/j.heliyon.2023.e20541 PMC10550503

[19] Gomaa, M. M. Interpretation of electrical properties for humid and saturated hematitic sandstone sample. *Presented at the 68th Conference and Exhibition incorporating SPE Europe: European Association of Geoscientists and Engineers (EAGE), Oral H021, Session Gravity, Magnetics, Mining and Geothermal, Opportunities in Mature Areas 4*. pp. 2182–2186. 10.3997/2214-4609.201402096 (2006).

[20] Gomaa, M. M. Relation between electric properties and water saturation for hematitic sandstone with frequency. *Ann. Geophys.* **51** (5/6), pp. 801–811. 10.4401/ag-3015 (2008).

[21] Kruschwitz, S., Binley, A., Lesmes, D. & Elshenawy, A. Textural controls on low-frequency electrical spectra of porous media. *Geophysics* **75** (4), WA113–WA123. 10.1190/1.3479835 (2010).

[22] Martinho, E., Almeida, F. & Matias, M. S. An experimental study of organic pollutant effects on time domain induced polarization measurements. *J. Appl. Geophys.* **60** (1), 27–40. 10.1016/j.jappgeo.2005.11.003 (2006).

[23] Revil, A., Schmutz, M. & Batzle, M. L. Influence of oil wettability upon spectral induced polarization of oil-bearing sands. *Geophysics* **76** (5), A31–A36. 10.1190/geo2011-0006.1 (2011).

[24] Sarkheil, H., Noughabi, K. S., Azimi, Y. & Rahbari, S. Fuzzy soil quality index using resistivity and induced polarization for contamination assessment in a lead and zinc drainage irrigation field study. *Ecol. Ind.* **152**, 110362. 10.1016/j.ecolind.2023.110362 (2023).

[25] Schmutz, M., Revil, A., Vaudelet, P., Batzle, M., Viñao, P. F. & Werkema D. D Influence of oil saturation upon spectral induced polarization of oil-bearing sands. *Geophys. J. Int.* **183** (1), 211–224. 10.1111/j.1365-246x.2010.04751.x (2010).

[26] Vanhala, H. Mapping oil-contaminated sand and till with the spectral induced polarization (sip) method. *Geophys. Prospect.* **45** (2), 303–326. 10.1046/j.1365-2478.1997.00338.x (1997).

[27] Vanhala, H., Soininen, H. & Kukkonen, I. Detecting organic chemical contaminants by spectral-induced polarization method in glacial till environment. *Geophysics* **57** (8), 1014–1017. 10.1190/1.1443312 (1992).

[28] Zhao, S., Zhang, B., Zhang, W., Su, X. & Sun, B. Impacts of contaminants from different sources on geotechnical properties of soils. *Sustainability* **15** (16), 12586. 10.3390/su151612586 (2023).

[29] Börner, F., Gruhne, M. & Schön, J. Contamination indications derived from electrical properties in the low frequency range. *Geophys. Prospect.* **41** (1), 83–98. 10.1111/j.1365-2478.1993.tb00566.x (1993).

[30] Chambers, J., Loke, M., Ogilvy, R. & Meldrum, P. Noninvasive monitoring of DNAPL migration through a saturated porous medium using electrical impedance tomography. *J. Contam. Hydrol.* **68** (1–2), 1–22. 10.1016/s0169-7722(03)00142-6 (2003). 10.1016/S0169-7722(03)00142-6 14698868

[31] Gamal, Z., Abdel Aal, E. A. & Atekwana Spectral induced polarization (SIP) response of biodegraded oil in porous media. *Geophys. J. Int.* **196**, 804–817. 10.1093/gji/ggt416 (2014).

[32] Olhoeft, G. R. Low-frequency electrical properties. *Geophysics* **50** (12), 2492–2503. 10.1190/1.1441880 (1985).

[33] Schmutz, M., Blondel, A. & Revil, A. Saturation dependence of the quadrature conductivity of oil-bearing sands. *Geophys. Res. Lett.* **39** (3). 10.1029/2011gl050474 (2012).

[34] Sun, H. et al. Detection and identification of oil spill species based on polarization information. *PLoS One*. **18** (11), e0291553. 10.1371/journal.pone.0291553 (2023). 38032948 10.1371/journal.pone.0291553 PMC10688671

[35] Titov, K., Kemna, A., Tarasov, A. & Vereecken, H. Induced polarization of unsaturated sands determined through time domain measurements. *Vadose Zone J.* **3** (4), 1160–1168. 10.2136/vzj2004.1160 (2004).

[36] Binley, A., Slater, L. D., Fukes, M. & Cassiani, G. Relationship between spectral induced polarization and hydraulic properties of saturated and unsaturated sandstone. *Water Resour. Res.* **41** (12). 10.1029/2005wr004202 (2005).

[37] Deng, Y. et al. Application of spectral induced polarization for characterizing surfactant-enhanced DNAPL remediation in laboratory column experiments. *J. Contam. Hydrol.* **230**, 103603. 10.1016/j.jconhyd.2020.103603 (2020). 31980237 10.1016/j.jconhyd.2020.103603

[38] Dong, Y., Xia, T., Meng, J. & Mao, D. Imaging LNAPL distribution at a former chemical plant with time-domain induced polarization. *Sci. Rep.* **14** (1). 10.1038/s41598-024-66782-8 (2024). 10.1038/s41598-024-66782-8 PMC11303548 39107372

[39] Slater, L. & D Near surface electrical characterization of hydraulic conductivity: from petrophysical properties to aquifer geometries - A review. *Surv. Geophys.* **28** (2–3), 169–197 (2007).

[40] Xia, T., Huisman, J. A., Chao, C., Li, J. & Mao, D. Induced polarization monitoring of in-situ chemical oxidation for quantification of contaminant consumption. *J. Contam. Hydrol.* **269**, 104481. 10.1016/j.jconhyd.2024.104481 (2024). 39647440 10.1016/j.jconhyd.2024.104481

[41] Scott, J. B. T. & Barker, R. D. Determining pore-throat size in Permo‐Triassic sandstones from low‐frequency electrical spectroscopy. *Geophys. Res. Lett.* **30** (9). 10.1029/2003gl016951 (2003).

[42] Garba, M. A., Vialle, S., Madadi, M., Gurevich, B. & Lebedev, M. Electrical formation factor of clean sand from laboratory measurements and digital rock physics. *Solid Earth*. **10** (5), 1505–1517. 10.5194/se-10-1505-2019 (2019).

[43] Gomaa, M. M., Elshenawy, A. M., Basheer, A. A., Moawad, M. & Kotb, A. Electrical properties of a dry mixture of sand and shale. *SEG Global Meeting Abstracts*. 299–302. 10.1190/iceg2021-076.1 (2021).

[44] Hui, K. S. et al. Experimental study on the electrical conductivity of quartz andesite at high temperature and high pressure: evidence of grain boundary transport. *Solid Earth*. **6** (3), 1037–1043. 10.5194/se-6-1037-2015 (2015).

[45] Nordsiek, S. & Weller, A. A new approach to fitting induced-polarization spectra. *Geophysics* **73** (6), F235–F245. 10.1190/1.2987412 (2008).

[46] Slater, L. D. & Lesmes, D. IP interpretation in environmental investigations. *Geophysics* **67** (1), 77–88. 10.1190/1.1451353 (2002a).

[47] Slater, L. & Lesmes, D. P. Electrical-hydraulic relationships observed for unconsolidated sediments. *Water Resour. Res.* **38** (10). 10.1029/2001wr001075 (2002).

[48] Titov, K., Tarasov, A., Ilyin, Y., Seleznev, N. & Boyd, A. Relationships between induced polarization relaxation time and hydraulic properties of sandstone. *Geophys. J. Int.* **180** (3), 1095–1106. 10.1111/j.1365-246x.2009.04465.x (2009).

[49] Wang, M. et al. Electrical conductivity of anhydrous and hydrous gabbroic melt under high temperature and high pressure: implications for the high-conductivity anomalies in the mid-ocean ridge region. *Solid Earth*. **14** (8), 847–858. 10.5194/se-14-847-2023 (2023).

[50] Zhang, Z., Kruschwitz, S., Weller, A. & Halisch, M. Enhanced pore space analysis by use of CT, MIP, NMR, and SIP. *Solid Earth*. **9** (6), 1225–1238. 10.5194/se-9-1225-2018 (2018).

[51] Gomaa, M. M., Elshenawy, A. M., Basheer, A. A., Moawad, M. & Kotb, A. Synthetic mixture of sand and shale: how conductor (shale) and saturation influence electrical characteristics. *Appl. Water Sci.* **13** (10). 10.1007/s13201-023-01981-8 (2023).

[52] Soliman, S. R., Salama, Y. F., El-Sayed, M. I., Abdel-Fattah, M. I. & Abd-Allah, Z. M. Assessment of mineralogical and geochemical composition of oligocene/eocene black shale deposits in Beni Suef Area, Egypt. *Adv. Mater. Sci. Eng.* 1–12. 10.1155/2022/1606431 (2022).

[53] Pelton, W. H., Ward, S. H., Hallof, P. G., Sill, W. R. & Nelson, P. H. Mineral discrimination and removal of inductive coupling with multifrequency IP. *Geophysics* **43** (3), 588–609. 10.1190/1.1440839 (1978).

[54] Revil, A. & Florsch, N. Determination of permeability from spectral induced polarization in granular media. *Geophys. J. Int.*10.1111/j.1365-246x.2010.04573.x (2010).

[55] Zimmermann, E. et al. A high-accuracy impedance spectrometer for measuring sediments with low polarizability. *Meas. Sci. Technol.* **19** (10), 105603. 10.1088/0957-0233/19/10/105603 (2008).

[56] Gomaa, M. M. Kaolinite under pressure at audio frequency range and its electrical features. *NRIAG J. Astron. Geophys.* **9** (1), 176–189. 10.1080/20909977.2020.1719341 (2020).

[57] Gomaa, M. M. Grain shape and texture effect on electrical characterization of semi-conductor semi-insulator mixture. In *Arabian Journal of Geosciences, Special Issue Geology of Africa, 9thand 10th Conferences, Egypt*, Vol. 14 (24), p. 2802. 10.1007/s12517-021-08517-x (2021).

[58] Shen, H., Huang, Y., Illman, W. A., Su, Y. & Miao, K. Migration behaviour of LNAPL in fractures filled with porous media: laboratory experiments and numerical simulations. *J. Contam. Hydrol.* **253**, 104118. 10.1016/j.jconhyd.2022.104118 (2022). 36563651 10.1016/j.jconhyd.2022.104118

[59] You, G., Feng, F., Zhang, J. & Zhang, J. A study on fracture propagation of hydraulic fracturing in oil shale reservoir under the synergistic effect of bedding weak Plane–Discrete fracture. *Processes* **13** (2), 362. 10.3390/pr13020362 (2025).

[60] Blondel, A., Schmutz, M., Franceschi, M., Tichané, F. & Carles, M. Temporal evolution of the geoelectrical response on a hydrocarbon contaminated site. *J. Appl. Geophys.* **103**, 161–171. 10.1016/j.jappgeo.2014.01.013 (2014).

[61] Desmond, M., Mavrogiannis, N. & Gagnon, Z. Maxwell-Wagner polarization and Frequency-Dependent injection at aqueous electrical interfaces. *Phys. Rev. Lett.* **109** (18). 10.1103/physrevlett.109.187602 (2012). 10.1103/PhysRevLett.109.187602 23215330

[62] Marshall, D. J. & Madden, T. R. Induced polarization, a study of its causes. *Geophysics* **24** (4), 790–816. 10.1190/1.1438659 (1959).

[63] Revil, A. & Glover, P. W. J. Theory of ionic-surface electrical conduction in porous media. *Phys. Rev. B*. **55** (3), 1757–1773. 10.1103/physrevb.55.1757 (1997).

[64] Lesmes, D. P. & Friedman, S. P. Relationships between the electrical and hydrogeological properties of rocks and soils. *Water Sci. Technol. Library* 87–128. 10.1007/1-4020-3102-5_4 (2005).

[65] Wang, C. & Slater, L. D. Extending accurate spectral induced polarization measurements into the kHz range: modelling and removal of errors from interactions between the parasitic capacitive coupling and the sample holder. *Geophys. J. Int.* **218** (2), 895–912. 10.1093/gji/ggz199 (2019).

[66] Surfer, V. Jun. 15.5.382 (64-bit). (Golden Sofware, LLC, 2018). (accessed 7 2018); https://www.goldensofware.com/products/surfer/

